# Glycine propionyl-L-carnitine produces enhanced anaerobic work capacity with reduced lactate accumulation in resistance trained males

**DOI:** 10.1186/1550-2783-6-9

**Published:** 2009-04-02

**Authors:** Patrick L Jacobs, Erica R Goldstein, Will Blackburn, Ihsan Orem, John J Hughes

**Affiliations:** 1 Department of Exercise Science and Health Promotion, Florida Atlantic University, Davie, FL 33314, USA

## Abstract

**Background:**

Recent research has indicated that short term administration of glycine propionyl-L-carnitine (GPLC) significantly elevates levels of nitric oxide metabolites at rest and in response to reactive hyperaemia. However, no scientific evidence exists that suggests such supplementation enhances exercise performance in healthy, trained individuals. The purpose of this study was to examine the effects of GPLC on the performance of repeated high intensity stationary cycle sprints with limited recovery periods in resistance trained male subjects.

**Methods:**

In a double-blind, placebo-controlled, cross-over design, twenty-four male resistance trained subjects (25.2 ± 3.6 years) participated in two test sessions separated by one week. Testing was performed 90 minutes following oral ingestion of either 4.5 grams GPLC or 4.5 grams cellulose (PL), in randomized order. The exercise testing protocol consisted of five 10-second Wingate cycle sprints separated by 1-minute active recovery periods. Peak (PP) and mean values (MP) of sprint power output and percent decrement of power (DEC) were determined per bout and standardized relative to body masss. Heart rate (HR) and blood lactate (LAC) were measured prior to, during and following the five sprint bouts.

**Results:**

Significant main effects (p < 0.001) were observed for sprint bout order in values of PP, MP, DEC, and HR. There were significant main effects detected for condition in PP and MP (p < 0.05), with values across the five sprint bouts 2.6 – 15% greater with GPLC. Significant statistical interactions were detected between bout order and condition for both PP and MP (p < 0.05). There was a significant main effect of condition for LAC, LAC values 15.7% lower 4 min post-exercise with GPLC (p = 0.09) and with GPLC resulting in 16.2% less LAC at 14 min post-exercise (p < 0.05).

**Conclusion:**

These findings indicate that short-term oral supplementation of GPLC can enhance peak power production in resistance trained males with significantly less LAC accumulation.

## Background

It is known that exercise hyperemia can provide a dramatic elevation of blood flow to specific active skeletal musculature, which also corresponds to metabolic demand [1]. There is an immediate and rapid increase in flow in response to a single muscle contraction, and the magnitude of the increased flow is directly related to the intensity of the contraction [2]. During heavy exercise, blood flow to local muscle tissue can be increased proportionally with exercise intensity to levels 25 – 50 times of those at rest [3]. This process is primarily a function of vasodilation of the arterioles (distal, proximal, and feed) and the pre-capillary sphincters, which is to a great degree induced by factors such as adenosine, carbon dioxide, and potassium, which are released in proportion to intensity of effort by adjacent muscle fibers during exercise [4]. The close coupling of muscular blood flow and exercise intensity supports the theory that further elevations in localized blood flow during exercise may, in some cases, result in increased peak work capacity and/or increased resistance to local muscle fatigue, thereby enhancing exercise performance.

The process of vasodilation as a primary component of exercise hyperemia involves mechanisms other than the aforementioned muscle metabolite induced vasodilatory mechanisms (adenosine, CO_2_, K^+^). For example, the initial increases of blood flow (first 1 – 2s) during exercise are now believed to be related to increased concentrations of acetylcholine as released by the motor end-plate during muscle activation [5]. Tschakovsky and Joyner [6] outlined several mechanisms believed to contribute to the secondary phase of vasodilation (3+ sec) including flow mediated mechanisms, the mechanical muscle pump, mechanically induced responses, muscle activation mechanisms, and red blood cell HbO_2 _desaturation mechanisms. Each of these mechanisms can be associated with different variations and intensities of exercise stresses. However, each of these distinct mechanisms shares the common function of initiating the synthesis of nitric oxide (NO).

Nitric oxide (NO) is a very short-lived, reactive gaseous nitrogen molecule that is involved in a variety of physiological functions. Approximately twenty years ago, it was revealed that NO was the endothelial factor responsible for regulating muscle tone of vascular structures, originally referred to as endothelial dependent relaxation factor (EDRF) several years prior. However, a viable means to manipulate this molecule has not been identified. Therefore, it is uncertain at this time what influence increased production of NO would have on cardiovascular functioning and/or resistance to local muscle fatigue.

Nitric oxide is synthesized in endothelial cells from arginine via enzymatic action of endothelium nitric oxide synthase. This molecule diffuses easily into the vascular smooth muscle where it binds to the enzyme guanylyl cyclase, which in turn catalyzes the phosphorylation of gunaosine-5-triphosphate (GTP) into cyclic gyanosine monophosphate (cGMP). Cyclic GMP serves as an important second messenger for many physiological functions, including relaxation of smooth vascular muscle. The amino acid, arginine, acts as a precursor to NO synthesis. Due to this role, a significant nutritional supplement market has developed for arginine-based products which supposedly enhance the production of NO. However, the current scientific evidence does not support the efficacy of these products. To a great degree, the success of this marketing has been based on evidence that direct infusion of arginine has been shown to induce significant levels of vasodilation [7], with enhanced hemodynamics [8] in healthy persons. However, controlled investigations have indicated that oral arginine supplementation did not have any effect on 1) peripheral resistance or cardiac output with a single 6 g dose [9] 2) endothelium-dependent vasodilation with intake of 7 g daily for three days [10], or 3) endothelial function in healthy persons after 28 days with 20 g arginine supplemented per day [11]. It has also been shown that the arginine levels in healthy persons are actually greater than what should theoretically be sufficient to activate endothelial NOS and thereby produce NO [12]. Thus, arginine based supplementation for improved NO synthesis is without scientific basis.

An oral carnitine compound, glycine propionyl-L-carnitine (GPLC), has recently been shown by Bloomer and associates to induce increased levels of plasma nitrates and nitrites (NOx) at rest in sedentary persons [8]. The same research group has also documented a dramatic elevation in NOx levels at rest and in response to occlusive hyperaemic testing in fifteen healthy resistance trained men after seven days supplementation with 4 g GPLC daily [13]. Following five minutes of upper arm occlusion with isometric hand gripping, the NOx levels were increased 16% and 17% over resting values with GPLC at three and 10 minutes post-occlusion, respectively, compared with 4% and 6% increases in NOx with placebo. These early findings suggest potential applications in clinical conditions or sports settings in which enhanced blood flow would be beneficial. However, there has been no examination of the effects of GPLC supplementation on physiological functioning or sports performance in exercise trained persons. Therefore, the present study was performed to examine the effects of short-term GPLC supplementation (4.5 g) on performance of repeated high-intensity cycle sprints and consequential lactate accumulation.

## Methods

### Research Participants

Thirty two male individuals volunteered to serve as research participants for this investigation. Inclusion criteria stipulated that all subjects were between the ages of 18 and 35 years and had participated in resistance training activities at least twice per week over the six-month period immediately prior to enrolment in this study. All testing procedures were verbally explained in detail and subjects provided written informed consent prior to participation, in accordance with the guidelines established by the Institutional Medical Sciences Subcommittee for the Protection of Human Subjects.

### Study Protocol

A double-blind, placebo-controlled, cross-over design was utilized in this investigation. Research participants completed two testing sessions seven days apart using the same testing protocol. Either GPLC or placebo (PL) was administered orally 90 minutes prior to each test, in randomized order. (See Supplementation Protocol Section).

Subjects were directed to continue the same general lifestyle patterns of exercise and nutritional intake during each seven-day period prior to the two exercise testing sessions. To verify the consistency of training and diet, the subjects were directed to complete a 7-day exercise log and a 3-day dietary recall (two week days and one weekend day) for each week prior to testing. The exercise log provided information regarding the volume (sets and reps) of resistance training relative to upper body, lower body, or total body structural movements. The dietary intake information was analyzed using ESHA Food Processor SQL dietary analysis software (ESHA Research, Salem, OR).

All research participants completed at least two familiarization trials prior to participating in the two testing sessions. The familiarization sessions followed the same general protocol but without full measurements of the actual exercise trials. On test days, participants were asked to report to the testing laboratory in the morning following a 12-hour period without food. They were also asked to refrain from vigorous exercise in the 24-hour period prior to testing. On arrival to the laboratory, the participants were provided with the respective supplement assigned for that session (GPLC or PL) and began a 90 minute resting period prior to testing.

### Supplementation Protocol

The two high intensity exercise trials were performed under two conditions, one with GPLC and one without. The study supplements (GPLC, PL) were provided by Jarrow Formulas (Los Angeles, CA) in 750 mg capsules, with six capsules equivalent to the 4.5 gram daily dose. The GPLC was the USP grade nutritional product, GlycoCarn™ (Sigma Ta Health Sciences, S.p.A., Rome, Italy), which consists of a molecular bonded form of glycine and propionyl-L-carnitine. The dosage of GPLC applied in this study is the same as that applied in previous research finding elevated NOx levels at rest and in response to occlusive hyperaemia [13]. The PL capsules were visually identical and contained 750 mg of cellulose. The supplement assignments were blinded to both the research participants and the study investigators. Subjects ingested the respective 4.5 gram supplement with 8 ounces of water approximately 90 minutes prior to testing.

### Testing Protocol

The assessment protocol consisted of five maximal effort 10-second cycle sprints performed with 1-minute active recovery periods between bouts. While Wingate type testing is typically performed using a single 30 second work period, repeated 10 second sprints have been used when testing exercise capacities similar to those required in relatively intense exercise. The sprints were performed using a Monarch 894E leg ergometer (Monarch, Varberb, Sweden) outfitted with pedal cages. The external resistance applied was equivalent to 7.5% of each subject's body mass. The testing protocol included a 10-minute warm-up period cycling on the test bike at a pace of 60 RPM, without external resistance. Following the warm-up period, subjects were directed to gradually increase the pace of their pedalling over several seconds until they reached a maximal pace of unloaded sprinting. At this point, with a verbal cadence, external resistance was applied thereby initiating a 10-second period of sprint testing and data collection. Verbal encouragement was provided by the investigators to continue sprinting at maximal pace throughout the 10-second bout. Subjects were directed to continue pedalling at a slower controlled pace during the 1-minute active recovery periods. With five seconds remaining in the recovery period, subjects were again directed to gradually increase their pedalling to a sprinting pace for the second sprint. This procedure was continued for a total of five 10-second sprint bouts.

Anaerobic power output of the sprints was determined using the SMI OptoSensor 2000 (Sports Medicine Industries, Inc., St. Cloud, Minn). Values of power output determined included peak power (PP) and mean power (MP) which in this case were the average values of power output during the first five seconds and total ten second period, respectively. The third power output measure was a value of power decrement (DEC) in which the difference in power output between the first and second five second periods are expressed as a percentage of the first.

Blood lactate levels were assessed using the Accutrend^® ^Lactate analyzer (Sports Resource Group, Inc., Pleasantville, NY). The analyzer was calibrated using the standard control solutions prior to each testing session. Lactate values were determined at rest and post-exercise at minutes four and fourteen. Heart rate was measured using a Polar HR monitor system with values assessed at rest, during the final 5 seconds of each sprint as well as four and fourteen minutes following completion of the fifth sprint. Thigh girth was assessed using a Gulick tape with circumferential measurements taken 15 mm superior to the patella. Thigh girth was measured at rest and four minutes following completion of the final sprint interval.

### Statistics

Two-way repeated measures ANOVAs were used to determine whether there were statistically significant differences between conditions (GPLC, PL) and across time. In the cases where significant main effects of condition or condition × time interactions were detected, single degree of freedom contrasts were used to determine condition effects at each bout order without adjustment of the acceptable level of significance. Net lactate accumulation relative to the power output of the sprints was calculated as the difference between the lactate measures at rest and those at 14 min divided by the average of the five MP values. Relative total power decrement was calculated for PP and MP as the relative difference between the first and last bout of each test condition. Values were compared between conditions with one-way ANOVA for repeated measures. In all cases, p-values less than 0.05 were accepted to determine statistical significance. All analyses were performed using SPSS, Version 16.

## Results

### Participants

Twenty four of the 32 recruited subjects completed both exercise trials. The study subjects were aged 25.2 ± 3.6 years with a mean body mass of 87.1 ± 14.5 kg and stature of 177.8 ± 6.9 cm. The 24 study subjects were confirmed to satisfy the inclusion criteria of consistent participation in resistance training during the six months prior to this study. Eight of the recruited subjects declined to participate in the research trial past the two familiarization test sessions. The intense nature of this exercise protocol appears to be related to the relatively high rate of attrition (25%). All statistical analyses are based on the data collected from the 24 subjects that completed both sprint test sessions. Planned sample size (32) was based on an estimated 10% dropout rate establishing a 0.75 level of power with a 0.25 predicted effect size. The reduced number of subjects limited statistical power to the 0.65 level, and is seen as a limitation of the present study as potential differences between conditions may not have been detected.

### Lifestyle Records

#### Dietary log data

Macronutrient intake values for both study conditions are presented in Table 1. Dietary intake data for protein (g), carbohydrates (g), and fats (g) as well as total calories were analyzed to determine daily averages which were compared between study conditions. Analysis indicated that there were no significant differences in these nutrient values for the three-day period preceding each of the two exercise trials.

**Table 1:** Nutritional recall information placebo GPLC

	**Placebo**	**GPLC**
**Protein (gr)**	179.8 ± 74.6	184.9 ± 75.7
% total cals	29%	30%

**Carbohydrates (gr)**	272.6 ± 145.1	254.4 ± 130.0
% total cals	44%	42%

**Fats (gr)**	73.8 ± 30.2	75.7 ± 32.6
% total cals	27%	28%

**Total Calories**	2482.2 ± 739.9	2434.1 ± 761.0

#### Exercise log data

The exercise training records provided information related to the volume of resistance training performed during the seven day supplementation period. Subjects were asked to record the number of sets and repetitions performed for each training exercise per session. Resistance training movements were classified, by investigators, based on upper versus lower extremity movements and based on compound versus single-joint exercises, thus establishing four exercise categories: upper extremity compound, upper extremity single-joint, lower extremity compound, and lower extremity single-joint. Table 2 provides a comparison of the training volume between placebo and GPLC conditions relative to the exercise categories. Analyses revealed no significant differences in the number of sets or repetitions between conditions in any of the four exercise categories (p > 0.05).

**Table 2:** Exercise training volume placebo GPLC

		**Placebo**	**GPLC**
**Upper Extremity**	Sets	38.5 ± 16.8	37.9 ± 17.8
	
**Compound Exercises**	Reps	383.0 ± 199.3	470.0 ± 371.9

**Upper Extremity**	Sets	34.0 ± 21.7	36.3 ± 24.7
	
**Single Joint Exercises**	Reps	414.6 ± 262.8	470.0 ± 371.9

**Lower Extremity**	Sets	9.7 ± 5.8	8.0 ± 5.9
	
**Compound Exercises**	Reps	81.5 ± 57.5	92.4 ± 127.1

**Lower Extremity**	Sets	10.0 ± 7.4	9.6 ± 9.0
	
**Single Joint Exercises**	Reps	111.2 ± 90.8	159.8 ± 260.8

### Power Output

**Figure 1:**
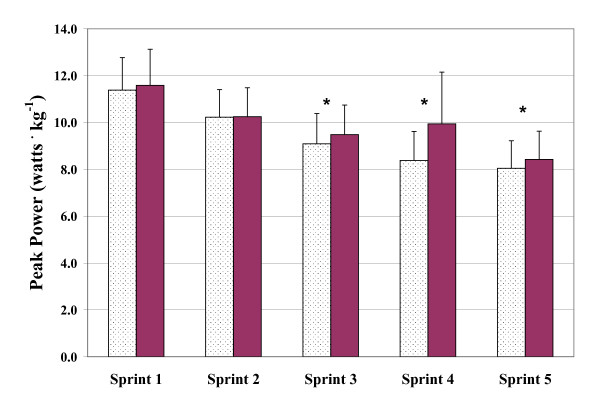
**Peak power (PP) determined during repeated cycling sprints with Placebo (dotted columns) and with GPLC (darkened columns)**. Note: Significant condition main effect (p < 0.01) and interaction effect (p < 0.05). Significant paired time contrasts for sprints 3, 4, and 5 (p < 0.05). Values are mean ± SD. * denotes statistically significant difference between conditions (p < 0.05)

**Figure 2:**
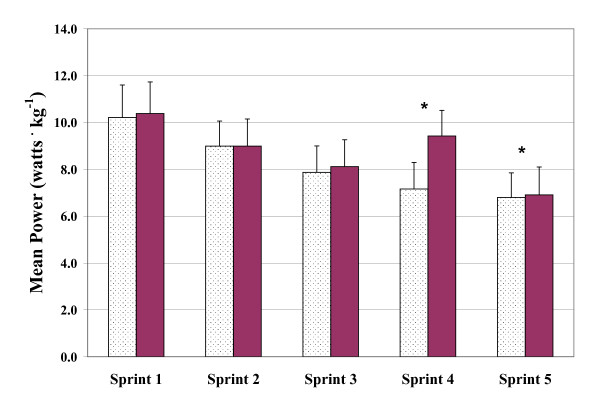
**Mean power (MP) determined during repeated cycling sprints with Placebo (dotted columns) and with GPLC (darkened columns)**. Note: Significant interaction effect (p < 0.05). Significant paired time contrasts for sprints 4 and 5 (p < 0.05). Values are mean ± SD. * denotes statistically significant difference between conditions (p < 0.05)

**Figure 3:**
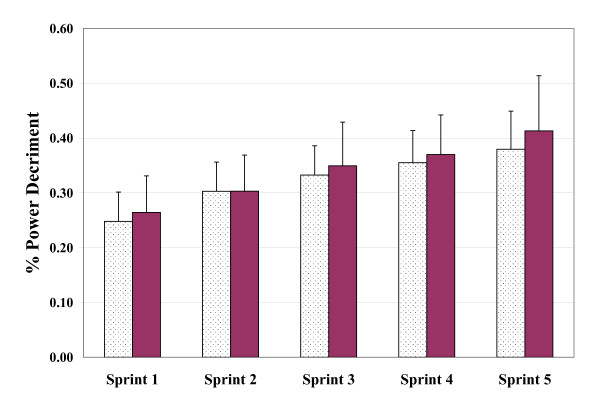
**Decrement in power output (DEC) determined during repeated cycling sprints with Placebo (dotted columns) and with GPLC (darkened columns)**. Note: No significant main condition or interaction effects (p > 0.05). Significant paired time contrast for sprint 5 (p < 0.05). Values are mean ± SD

**Figure 4:**
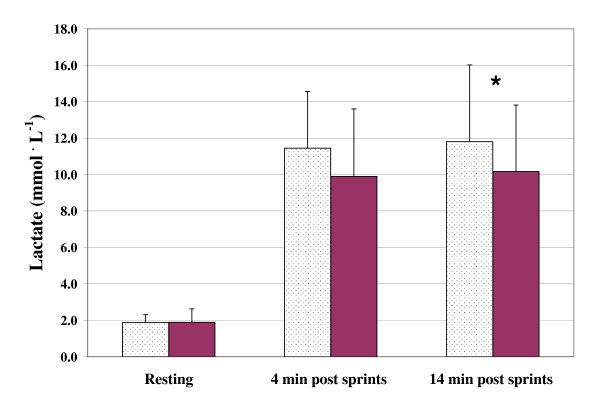
**Lactate (PP) assessed during at rest and 4 min and 14 min following repeated cycling sprints with Placebo (dotted columns) and with GPLC (darkened columns)**. Note: Significant condition main effect (p < 0.05). Significant paired time contrast for 14 min post sprints (p < 0.05) but not 4 min post sprint (p = 0.09). * denotes statistically significant difference between conditions (p < 0.05)

**Figure 5:**
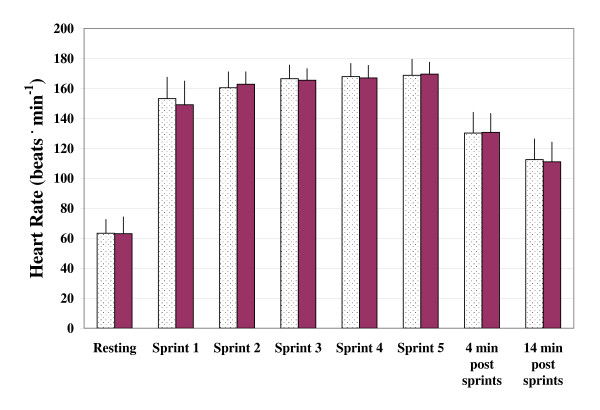
**Heart rate (HR) assessed at rest, during and following repeated cycling sprints with Placebo (dotted columns) and with GPLC (darkened columns)**. Values are mean ± SD.

The three measures of power output (PP, MP, and DEC) were found to vary significantly with bout order (p < 0.001). In the case of PP and MP, values decreased while DEC increased with subsequent sprint bouts. Mean values of PP, MP, and DEC across the five sprint bouts are presented graphically in Figures 1, 2 and 3, respectively. Figures 4 and 5 depict the HR and LAC responses across the five sprint bouts, again values increasing with the subsequent bouts.

#### Peak Power

Supplementation of GPLC had a significant main effect on PP (p < 0.05). Across the five sprint bouts, PP was 1.7%, 0.2%, 4.1%, 15.7%, and 4.4% greater with GPLC. There was also a significant interaction between GPLC and sprint bouts on PP. Analysis revealed that values of PP for bouts three, four and five were statistically greater (p's < 0.05) with GPLC.

#### Mean Power

There wasn't a statistically significant effect of GPLC on MP (p = 0.083). Mean values of MP were 2 – 24% greater with GPLC across sprint bouts one through five. A significant interaction was detected between GPLC and sprint bouts (p < 0.01). Post hoc one-way ANOVA for repeated measures showed MP produced during sprints four and five with GPLC were significantly greater than the values produced with PL (p's < 0.05).

#### Power Decrement

Figure 3 displays the DEC values during both test conditions. As previously mentioned, DEC increased significantly with ongoing sprint bouts. However, analysis of the DEC data did not reveal significant effects of GPLC (p = 0.65) or significant interaction with sprint bouts (p = 0.51). Interestingly, the difference between conditions in mean values of DEC tended to increase as sprint bouts progressed with a statistically significant difference (p < 0.05) in the fifth sprint with a 38% power decrement with PL while GPLC produced a 41.3% rate of power decrement.

Relative total power decrement within each test session for PP was lower with GPLC than PL, with 26.6% and 32.8% declines in those values respectively, however this difference was not statistically significant (p = 0.09). The mean MP total power decrement values were not statistically different between groups (p = 0.32) with 36.4% and 33.1% for GPLC and PL, respectively.

### Lactate

A significant main effect for condition was observed for lactate measures (p < 0.05). Figure 4 displays the lactate measures at rest as well as four and 14 minutes post-exercise. There were no significant differences between conditions in lactate levels at rest. Lactate measures taken at four and fourteen minutes post-exercise were 15.6% and 16.2% lower, respectively, with GPLC. Paired timepoint analyses indicated that the differences between conditions were statistically significant at 14 minutes post-exercise (p < 0.05) but not four minutes following the sprint bouts (p = 0.09). Net lactate accumulation per unit power output, calculated as (LAC_14_-LAC_rest_)^. ^(MP_ave_)^-1 ^differed significantly between conditions (p < 0.05). GPLC produced 22.8% less net lactate per watt than placebo, 0.947 and 1.227 mmol^. ^watt^-1^, respectively

### Heart rate

Heart rate was recorded at rest, during the final 10 seconds of each sprint bout, as well as 4 and 14 minutes post-exercise (see Figure 5). There were no significant effects of condition or interaction effects detected for values of HR. As previously mentioned, HR tended to increase across time with a considerable increase in HR from rest to bout 1, then slightly increasing with subsequent sprint bouts to peak values of approximately 169 bpm in both conditions. Post-exercise HR responses did not differ appreciably between the GPLC and PL conditions with values of approximately 130 and 111 bpm at four and 14 minutes, respectively, following the sprints.

### Thigh girth

There were no significant main condition effects or condition × time interactions in the measures of thigh girth. There was a significant main effect of time (pre-, post-exercise) indicating similar increases in thigh girth in both conditions (GPLC, PL). Girth increased from 57.1 ± 6.0 to 58.9 ± 6.1 cm for the PL condition with the GPLC condition producing a slightly greater (5%) expansion of thigh circumference from 57.3 ± 6.4 cm at rest to 59.1 ± 6.3 cm four min after exercise.

## Discussion

The findings of this study demonstrate that short-term GPLC supplementation may significantly enhance anaerobic work capacity in resistance trained males. These findings are particularly striking when considered in combination with the significant reduction in lactate accumulation following GPLC supplementation. A post-hoc analysis revealed a 22.8% reduction in the ratio of net lactate accumulation per unit of power output. The effects documented in this investigation generally exceed those of previous studies investigating L-carnitine supplementation and exercise performance. In order to discuss the findings of the present study in reference to previous work, it is useful to first consider the known metabolic functions of carnitine and its acyl variations.

Carnitine is endogenously metabolized and obtained from dietary sources such as meat and dairy products. Over 80% of carnitine is found in skeletal muscle tissue where it fulfils two vital metabolic functions. Both functions involve the exchange of activated acyl carboxylic acids (acyl groups) between carnitine and Coenzyme A.



The total carnitine pool is composed of free carnitine and acylcarntines (both long chain and short chain) and the balance between free carnitine and the acyl variations is an indication of metabolic activity and exercise intensity.

The metabolic function commonly associated with carnitine is the shuttling of free fatty acids (long chain acyl-CoAs) into the inner region of the mitochonia where beta oxidation of the acyl groups takes place. The carnitine pool provides a vital role in this process as the long chain fatty acyl-CoAs are actually unable to enter the inner mitochondrial matrix due to their large size. Acyl groups are exchanged between free carnitine and acylcarnitine, which is readily able to travel into the inner matrix where the acyl-CoA is reformed using the reverse mechanism. The process of conversion between free carnitine and acylcarnitines is dependent on three carnitine enzymes. Carnitine Palmitoyltransferase (CPT1) activates the conversion of carnitine and long chain acyl-CoAs to form long chain acetylcarnitine (most often acetylcarnitine) and Coenzyme A which can effectively pass into the inner regions of the mitochondia. CPT1 activity is based on adequate muscle levels of carnitine, which progressively declines with increased production of acetylcarnitines as exercise intensity and/or duration increases. Thus, CPT1 is considered the rate limiting enzyme of oxidation of long chain fatty CoAs with muscle carnitine levels actually serving as a control mechanism of this metabolic pathway. The association of muscle carnitine levels and acyl-CoA oxidation is the basis of a multi-million energy and weight loss nutraceutical industry.

Carnitine serves a second critical metabolic function, buffering the Coenzyme A pool against accumulation of short chain acetyl-CoAs, which at increasing levels reduce the activity of the pyuvate dehydrogenase complex (PDC). Increased levels of acetyl-CoAs inhibit PDC activity thereby reducing the ability to produce a substrate capable of entering the citric acid cycle thereby resulting in increased lactate production. The shift from short chain acetyl-CoA to lactate production is considered an indication that anaerobic processes exceed the capability of the citric acid cycle. In the setting of increased short chain acetyl-CoAs, carnitine is capable of accepting the acyl group in the development of acylcarnitine (generally acetylcarnitine) effectively reducing the level of acetyl-CoA and extending the ability to continue high intensity exercise. This process is limited by the muscle carnitine levels which are gradually reduced with continued intense exercise. Thus, muscle carnitine levels have been associated with the ability to sustain high anaerobic efforts with reduced output of lactate. Another multi-million dollar industry, based on enhancement of sports performance, is predicated on these anaerobic buffering processes and the role of carnitine.

Investigations of the effects of L-carnitine supplementation and exercise performance have yielded equivocal findings which have been carefully discussed in several published reviews [9,14,15]. The majority of exercise trials examining the efficacy of L-carnitine have based their work on the role of carnitine in the transport of fatty acids and therefore used endurance performance protocols with outcomes measures including maximal oxygen uptake (VO_2 max_) or markers of anaerobic threshold as determined during graded incremental exercise testing. In general, most studies have failed to document increases in VO_2 max _or performance markers whether examining untrained or athletic persons. The authors of those individual studies as well as the reviewers have generally attributed the lack of performance benefits with L-carnitine to the inability to increase resting muscle carnitine concentrations. However, several studies have reported increased VO_2 max _[12,16,17] and/or reduced post-exercise lactate accumulation [17,18]. While there have been positive reports of carnitine supplementation and enhanced exercise performance and/or improved responses to exercise, there has been a general consensus to disregard the validity of those findings as the predominate opinion is that any performance enhancements must be predicated on increased resting muscle carnitine levels. Thus, there has been a general reconsideration of carnitine supplementation has a means not to improve exercise performance but rather to enhance recovery from hypoxic stresses associated with exercise [19,20].

Recently, it has been shown that muscle carnitine content can be increased via an interesting approach. Stephens and associates [21] reported that infusion of a physiologically high level of insulin concurrent with infusion of carnitine significantly elevated the muscle carnitine concentrations in eight healthy men. Moreover using the same hyperinsulinemia strategy, that research group also documented reduced PDC activity and muscle lactate levels with increased muscle glycogen stores presumably related to increased muscle carnitine levels following IV infusion of insulin and carnitine [22]. These findings are clear evidence that it is possible to increase muscle carnitine levels, in this case via the influences of high insulin levels. It is well established that insulin itself acts as a regulator for vasodilation and blood flow by modulating nitric oxide synthesis and release [23]. Thus, it is possible that the increase in muscle carnitine levels were increased to a great extent due to NO providing vasodilation and enhanced capillary filling, which provides direct muscle access to the elevated plasma concentration of carnitine. Stephens et al. [21,22] suggested their findings may provide insight into persons with diabetes and obesity where fat oxidation processes are limited, it is doubtful this approach would be beneficial in those clinical populations. Rather, those clinical conditions are commonly associated with varying states of insulin resistance which would likely limit the effectiveness of this carnitine loading strategy.

The research of Arenas et al. [24,25] and Huertes et al. [26] provides an alternative perspective to the application of carnitine loading for supraphysiological resting concentrations. Those researchers examined the application of L-carnitine (1–2 grams daily) in long distance runners and sprinters over one to six month periods of training. They documented reductions in free carnitine with intense training in agreement with the previous work of other researchers but provided the unique finding that carnitine supplementation alleviated all training induced deficits in total and free carnitine. Increased activity of respiratory chain enzymes and PDH activity were associated with increased VO_2 max _in the supplemented athletes. Thus, these findings would suggest that chronic carnitine administration may replenish gradual chronic reductions in resting muscle carnitine levels, as developed with ongoing stressful exercise training. In this way it is not necessary to attain considerably increased levels of muscle carnitine to effectively enhance performance, but rather prevent deleterious reductions in those concentrations. A means to apply this approach to high intensity exercise, where reduced free carnitine supply is associated with anaerobic work capacity and resistance to local muscle fatigue, would provide benefits to many different populations ranging from clinical populations with neuromuscular disorders to elite athletic competitors.

The acylcarnitine pool includes the previously mentioned acetylcarnitine and propionylcarnitine (PC), which is metabolized by carnitine acetyltransferase from propionyl-CoA. Propionylcarnitine possesses three characteristics that distinguish this acylcarnitine from other members of the carnitine pool. First, it has a unique vasodilatory effect which is specific to this compound. This may be the reason that PC has been shown to have a high affinity for both skeletal and cardiac muscle tissue. Secondly, PC provides a source of propionyl units which are easily transformed into succinate for mitochondrial utilization in the citric acid cycle as a source of anaplerotic energy. In this way, PC supplies an active energy substrate even during periods of limitations in localized oxygen availability, ie muscle ischemia. Finally, PC provides a replenishment of free carnitine in cases of deficiency with intense exercise or disease. Propionyl-L-carnitine, being a prescription medication in both Europe and the United States, has been examined primarily as a treatment in clinical populations with apparent muscle carnitine deficiencies. Controlled clinical trials indicated that PLC provides enhanced work capacity in persons with congestive heart failure [27] and peripheral vascular disease [28].

Glycine propionyl-L-carnitine (GPLC) is a novel nutrient consisting of a molecularly bonded combination of PLC and the amino acid glycine. Glycine is considered as a glucogenic amino acid in that it helps to regulate blood sugar levels and is also very important in the formation of creatine. Interestingly, glycine has been shown to have its own independent vasodilatory effects [29]. Limited research has examined the effects of GPLC on exercise performance within the general population or athletes. An ishchemic-reperfusion model was used by Bloomer, Smith, and Fisher-Wellman to examine blood nitrite/nitrate levels as an indication of NO production [13]. This model provides a means to assess physiological measures such as blood flow and increased levels of NO in response to occlusive stresses similar to those exhibited during high intensity resistance training. Those studies indicated that GPLC supplementation at 4.5 g per day for one week produced dramatically greater blood nitrite/nitrate levels both at rest and in response to the occlusion/reperfusion stress. Those findings are particularly notable as GPLC is the first and only nutritional supplement product proven to increase NO synthesis. Smith and associates [30] reported findings related to a group of previously inactive persons, who for eight weeks performed stationary cycling and/or walking with GPLC supplementation. Study participants were randomized to receive placebo, 1 or 3 g GPLC per day. The exercise testing, performed prior to and following the eight weeks of training, consisted of the standard Bruce protocol treadmill test and standard 30 sec Wingate test. Thus, the testing procedures introduced a high degree of variability which may have limited measurable performance effects with GPLC. However, there was a significant treatment main effect with the GPLC 1 g/day group displaying a greater lactate threshold than the placebo group (64.5% vs 56. 0% VO_2 max_). There was also a statistically non-significant trend (p = 0.09) for greater relative change in lactate threshold in both GPLC groups (1 g, 10.3%; 3 g, 8.8%) compared with the placebo group (3.5%). There was no difference in muscle carnitine measures between study groups following eight weeks of supplementation.

The results of the present investigation do not directly conflict with the findings of the Webb et al. study. The testing protocol used in the present study differed substantially from the graded incremental treadmill protocol used in the Webb report. However, an increased work capacity to lactate threshold was associated with GPLC in those treadmill assessments. The reported lack of anaerobic benefits of GPLC in the Webb study was based on performance of a single 30-sec Wingate sprint. The present investigation applied repeated 10-sec sprints, and found no significant differences between groups in the first two sprints. It was only during the third, fourth, and fifth sprints that the GPLC condition produced significantly more power output and with less lactate accumulation.

It is possible to establish a plausible mechanistic explanation using 1) the performance outcomes of the present investigation in combination with 2) previously established mechanisms of the underlying carnitine molecules, and 3) recent reports of increased muscle carnitine levels via insulin infusion. The authors of the present study propose that GPLC provides theoretical advantages by way of replenishment of carnitine stores which generally decline during stressful exercise and the inclusion of an additional energy source, via characteristics that are unique to this molecularly bonded form of carnitine. First, the vasodilatory effects associated with increased NO are seen as the critical action responsible for these impressive findings. Prior studies have generally indicated that L-carnitine does not provide performance benefits, which usually was attributed to the inability to significantly increase resting muscle carnitine concentrations. The exception to that rule has been with increased insulin levels which are known to modulate the NO pathway. It is proposed that GPLC provides a means to elevate blood flow during vigorous exercise via increased production of NO. Reduced vasotension and relaxed capillary sphincters allow considerably elevated local blood flow into the capillary bed thereby providing an enhanced exchange of nutrients and metabolic products. The walls of capillaries are composed of a single layer of endothelium cells without the smooth musculature found in terminal arterioles. Capillaries are surrounded by several muscle fibers within the same motor unit thereby providing direct interface with the blood system and the nutrients it carries.

Within the muscle tissue, the supplemented acylcarntitines are converted to free carnitine and propinyl-CoA. Propionate serves as an anaplerotic energy substrate even in the environment of muscle ischemia evident with intense muscular exertion or disease states. Free carnitine is also produced via this mechanism thereby replenishing, to some degree, muscle carnitine levels that tend to decline with increasing conversion to long chain acylcarnitines during transport of acyl-CoAs into the mitochondrial matrix. Deficits in carnitine stores exhibited during high intensity anaerobic work may be reduced as replenishing free carnitine levels facilitates the production of short chain acylcarnitines as a buffering process that reduces lactate accumulation. This model may provide enhanced fatty acid oxidation at rest and during submaximal exercise to the point of lactate threshold. Complementary anaerobic benefits are provided with high intensity exercise via enhanced blood flow related to increased NO synthesis, the addition of an anaplerotic energy source in propionate. Anaerobic power is enhanced by buffering Coenzyme A by carnitine thereby preventing the elevation of Acetyl-CoA levels which would generally hinder the activity of the PDC thereby stimulating the production of lactate. Thus, at rest and during moderate intensity exercise GPLC appears to enhance fatty acid oxidation and aerobic metabolism while it increases anaerobic power with reduced lactate production during high intensity exercise.

This simplistic mechanistic model is based on numerous previously established functions of the total carnitine pool, in conjunction with the unique characteristics of GPLC as reported in recent investigations, as well as from the present study. The 4.5 gram dosage of GPLC used in this study was similar to that applied by Bloomer [13], but that study applied the daily dose over a one week period. Furthermore, the present study did not measure NOx, thus it is not possible to establish the role of NO in the findings of the present study. In fact, the only means of assessing reactive hyperemia of the lower extremities in the present study was the thigh girth as determined using a basic Gulick measuring tape. Based on the magnitude of NOx increases reported by Bloomer's group, it was hypothesized that GPLC may produce increases in local blood flow which might be measurable using a basic girth assessment. However, the increase in thigh girth was not significantly different between study conditions. Thus, it is uncertain whether the performance benefits observed in the present study were related to increased levels of NO or other mechanisms of action. Certainly, the present investigation should be replicated, with examination of varying dosages over extended periods of time, with valid outcome measures that indicate critical metabolic pathway activity. The present study is seen as proof of concept that oral GPLC administration can increase peak anaerobic power output with reduced lactate accumulation.

## Conclusion

In conclusion, the findings of the present investigation demonstrate that short-term supplementation of GPLC produces significant enhancement of anaerobic power during repeated cycle sprints in resistance trained men. The increase in peak power output was accompanied by a significantly lower accumulation of lactate. These findings provide the first evidence that the previously observed increases in NO with GPLC may be associated with performance improvements in trained individuals. While the present findings should be limited at this time to the resistance trained male population under direct examination, these results suggest application in various groups that exhibit reduced muscle carnitine content and the associated limitations in physical performance.

A simple theoretical model of GPLC and altered metabolic activity has been presented. These authors suggest that the vasodilatory effects of GPLC, presumably associated with increased NO synthesis, allow an effective interface between muscle tissue and the blood stream as the capillary bed progressively engorges during high intensity exercise. Thus, a paradigm shift from the conventional approach of nutritional supplementation has been established. It has been generally assumed that resting nutrient stores must be significantly increased in order to produce performance enhancements. It is suggested that, in some situations, certain nutrients that are utilized in the metabolic activities of high intensity exercise may be effectively restored via diffusion from higher concentrations of that nutrient within the blood serum. The effectiveness of this general strategy has been demonstrated previously with different micronutrients via infusion of insulin and ingestion of high glycemic index carbohydrate foods to induce spikes of insulin. This is, to some degree, the very basis of various nutrient timing strategies commonly applied in athletic training. It appears that GPLC, in conjunction with high intensity exercise, has the capacity to effectively enhance the uptake of certain micronutrients into muscle tissue thereby providing a viable alternative for the low-carbohydrate lifestyle and for persons with reduced insulin sensitivity.

## Competing interests

The authors declare that they have no competing interests.

## Authors' contributions

PJ was responsible for study design, data collection, statistical analysis, and manuscript preparation. EG was responsible for data input and analysis as well as manuscript preparation. WB carried out data collection and input. IO carried out literature review, data collection and input. JH was responsible for data analysis and manuscript preparation.

## References

[B1] Hamman JJ, Kluess HA, Buckwalter JB, Clifford PS (2005). Blood flow response to muscle contractions is more closely related to metabolic rate than contractile work. J Appl Physiol.

[B2] Naik JS, Valic Z, Buckwalter JB, Clifford PS (1999). Rapid vasodilation in response to a brief titanic muscle contraction. J Appl Physiol.

[B3] Anderson P, Saltin B (1985). Maximal perfusion of skeletal muscle in man. J Physiol-London.

[B4] Haddy FJ, Scott JB (1975). Metabolic factors in peripheral circulatory regulation. Fed Proc.

[B5] Kurjiaka DT, Segal SS (1995). Conducted vasodilation elevates flow in arteriole networks of hamster striated muscle. Am J Physiol.

[B6] Tschakovsky ME, Joyner MJ (2007). Nitric oxide and muscle blood flow in exercise. Appl Physiol Nutr Metab.

[B7] Hishikawa K, Nakaki T, Tsuda M, Esumi H, Oshima H (1992). Effects of systemic L-arginine administration on hemodynamics and nitric oxide release in man. Jpn Heart J.

[B8] Bode-Boger SM, Boger RH, Galland A, Tsikas D, Frolich J (1998). L-arginine-induced vasodilation in healthy humans: pharmacokinetic-pharmacodymanic relationship. Br J Clin Pharmacol.

[B9] Brass EP (2000). Supplemental carnitine and exercise. Am J Clin Nutr.

[B10] Adams MR, Forsyth CJ, Jessup W, Robinson J, Celermajer DS (1995). Oral arginine inhibits platelet aggregation but does not enhance endothelium-dependent dilation in healthy young men. J Amer Col Cardiology.

[B11] Chin-Dusting JP, Alexander CT, Arnold PJ, Hodgson WC, Lux AS, Jennings GL (1996). Effects of in vivo and in vitro L-arginine supplementation on healthy human vessels. J Cardiovasc Pharmacol.

[B12] Marconi C, Sessi G, Carpinelli A, Cerretelli P (1985). Effects of L-carnitine loading on the aerobic and anaerobic performance of endurance athletes. Eur J Appl Physiol.

[B13] Bloomer RJ, Smith WA, Fisher-Wellman KH (2007). Glycine propionyl-L-carnitine increases plasma nitrate/nitrite in resistance trained men. J Int Soc Sports Nutr.

[B14] Brass EP, Hiatt WR (1998). The role of carnitine and carnitine supplementation during exercise in man and in individuals with special needs. J Am Coll Nutr.

[B15] Heinonen OJ (1996). Carnitine and physical exercise. Sports Med.

[B16] Dragan GI, Vasiliu A, Georgescu E, Dumas I (1987). Studies concerning chronic and acute effects of L-carnitine on some biological parameters in elite athletes. Physiologie.

[B17] Vecchiet L, Di Lisa F, Pieralisi G (1990). Influence of L-carnitine supplementation on maximal exercise. Eur J Appl Physiol.

[B18] Siliprandi N, Di Lisa F, Pieralisi G (1990). Metabolic changes induced by maximal exercise in human subjects following L-carnitine administration. Biochim Biophys Acta.

[B19] Bloomer RJ (2007). The role of nutritional supplements in the prevention and treatment of resistance exercise-induced skeletal muscle injury. Sports Med.

[B20] Kraemer WJ, Volek JS, Dunn-Lewis C (2008). L-carnitine supplementation: Influence upon physiological function. Curr Sports Med Rep.

[B21] Stephens FB, Constantin-Teodosiu D, Laithwaite D, Simpson EJ, Greenhaff PL (2005). Insulin stimulates L-carnitine accumulation in human skeletal muscle. FASEB J.

[B22] Stephens FB, Constantin-Teodosiu D, Laithwaite D, Simpson EJ, Greenhaff PL (2006). An acute increase in skeletal muscle carnitine content alters fuel metabolism in resting human skeletal muscle. J Clin EndoMetab.

[B23] Steinberg HO, Brechtel G, Johnson A, Fineberg N, Baron AD (1994). Insulin-mediated skeletal muscle vasodilation is nitric oxide dependent. A novel action of insulin to increase nitric oxide release. Clin Invest.

[B24] Arenas J, Huertas R, Campos Y, Diaz E (1992). Respiratory chain enzymes in muscle of endurance athletes: effect of L-carnitine. Biochem Biophys Res Commun.

[B25] Arenas J, Ricox JR, Encinas AR, Pola P (1994). Effects of L-carnitine on the pyruvate dehydrogenase complex and carnitine palmitoyl transferase activities in muscle of endurance athletes. FEBS Lett.

[B26] Huertas R, Campos Y, Diaz E (1992). Respiratory chain enzymes in muscle of endurance athletes: effect of L-carnitine. Biochem Biophys Res Commun.

[B27] Anand I, Chandrashekhan Y, De Guili F, Pasini E (1998). Acute and chronic effects of propionyl-L-carnitine on the hemodynamics, exercise capacity, and hormones in patients with congestive heart failure. Cardiovasc Drugs Ther.

[B28] Dal Lago A, De Martini D, Flore R (1999). Effects of propionyl-L-carnitine on peripheral arterial obliterative disease of the lower limbs: a double-blind clinical trial. Drugs Exp Clin Res.

[B29] Podoprigora GI, Nartsissov YR, Aleksandrov PN (2005). Effect of glycine on microcirculation in pial vessels of rat brain. Bull Exp Biol Med.

[B30] Smith WA, Fry AC, Tschume LC, Bloomer RJ (2008). Effect of glycine propionyl-L-carnitine on aerobic and anaerobic performance. Int J Sport Nutr Exerc Metab.

